# Supplementary material for: Mycobiome Study Reveals Different Pathogens of Vulvovaginal Candidiasis Shape Characteristic Vaginal Bacteriome
Source: Microbiol Spectr. 2023 Mar 30;11(3):e03152-22. doi: 10.1128/spectrum.03152-22 (PMC10269694; doi:10.1128/spectrum.03152-22)
Supplement: Supplemental file 1 — Supplemental material. Download spectrum.03152-22-s0001.pdf, PDF file, 0.6 MB [file spectrum.03152-22-s0001.pdf]

Supplemental Figure 1

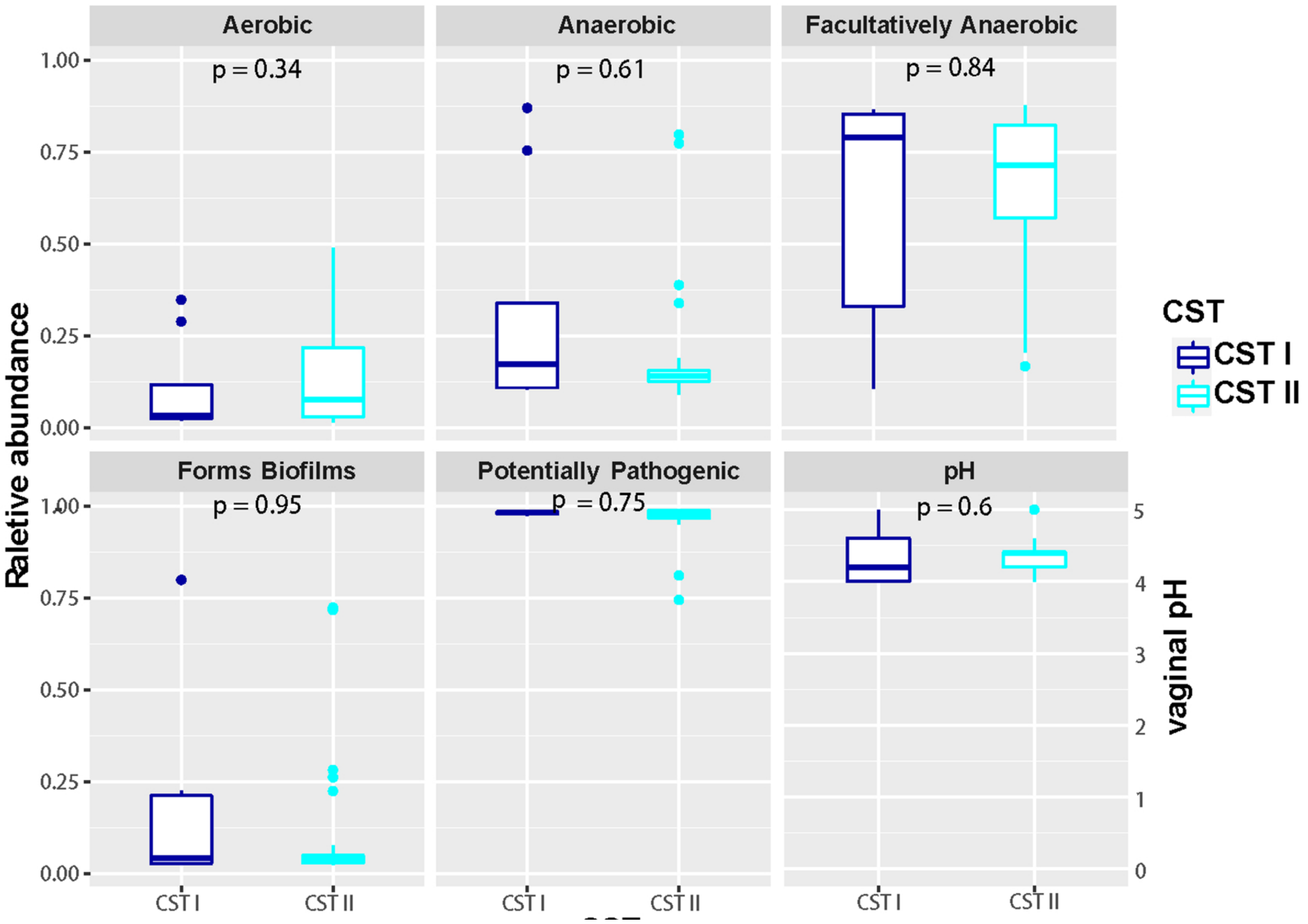

FIG S1：Relative abundance of each predictive organism-level microbial phenotypes at different CST classes.

The X-axis refers to predictive phenotypes, and the Y-axis demonstrates the relative abundances of the phenotypes. P value represents the results of the Wilcoxon rank-sum test between different CST classes.

Supplemental Figure 2

A

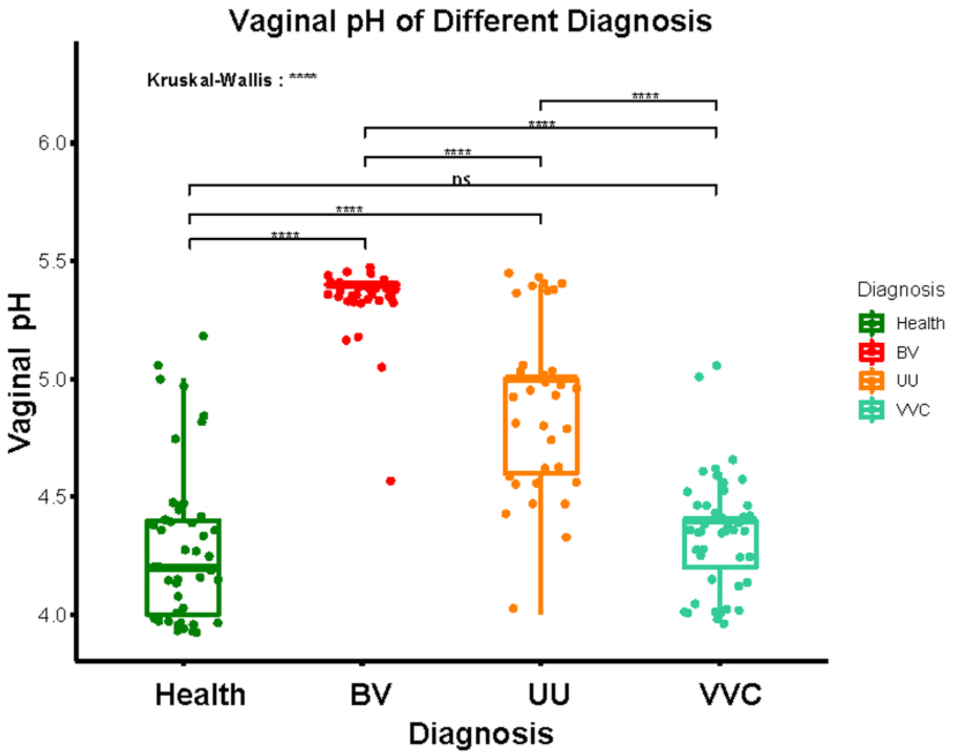

B

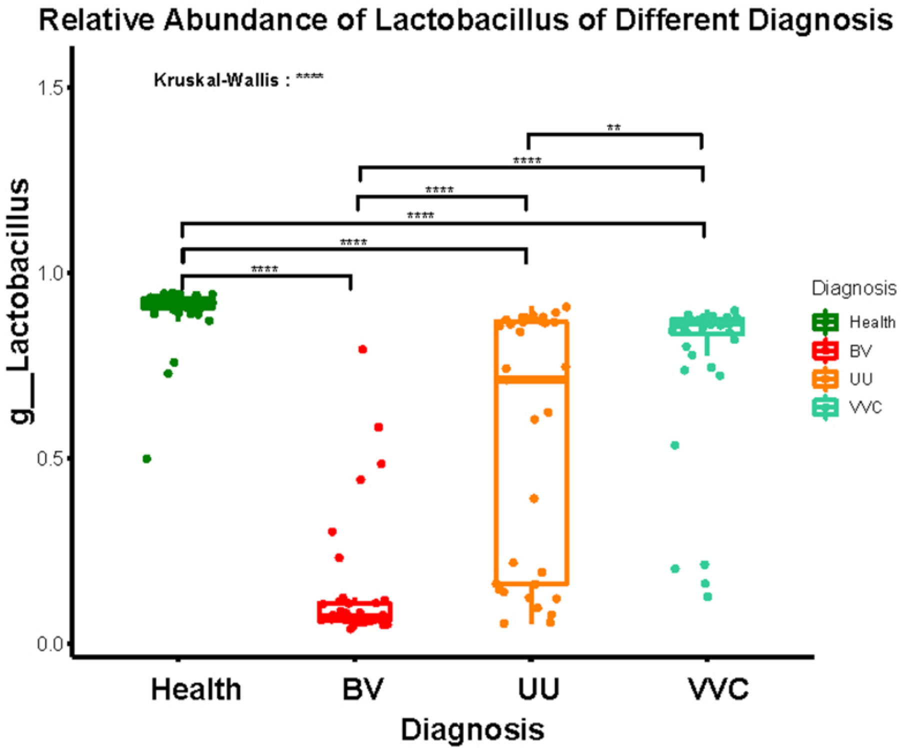

FIG S2: Vaginal pH levels and lactobacillus genus abundance values among health, BV, UU and VVC groups.

Vaginal pH levels and lactobacillus genus abundance values among health, BV, UU and VVC groups. Multi-group samples were compared by Kraskal-Wallis test, and further comparisons between two groups were test by wilcoxon test.

"ns" means $p > 0.05$, "**" means $p < 0.01$, "****" means $p < 0.0001$.

Supplemental Table 1

Diagnosis	Explanation
BV	bacterial vaginitis
VVC	vulvovaginal candidiasis
UU	vaginitis caused by *Ureaplasma urealyticum*

Clinical metadata of subjects recruited into this study.

Sample ID	Diagnosis	Age	vaginal cleaning degree	Vaginal pH
N1	health	36	II	4.4
N2	health	46	II	4.2

N3	health	30	II	5.0
N4	health	45	II	4.2
N5	health	42	II	4.4
N6	health	30	II	4.4
N7	health	30	II	4.4
N8	health	26	II	4.0
N9	health	53	II	5.0
N10	health	28	II	4.0
N11	health	39	II	4.8
N12	health	37	II	4.2
N13	health	40	II	4.2
N14	health	34	II	5.0
N15	health	39	II	4.8
N16	health	21	II	4.0
N17	health	31	II	4.0
N18	health	30	II	4.4
N19	health	36	II	5.2
N20	health	31	II	4.2
N21	health	32	II	4.4
N22	health	30	II	4.0
N23	health	31	II	4.4
N24	health	32	II	4.2
N25	health	33	II	4.4
N26	health	49	II	4.0
N27	health	55	II	4.2
N28	health	42	II	4.0

N29	health	32	II	4.8
N30	health	34	II	4.2
N31	health	46	II	4.2
N32	health	34	II	4.4
N33	health	50	II	4.4
N34	health	45	II	4.4
N35	health	34	II	4.4
N36	health	51	II	4.0
N37	health	35	II	4.0
N38	health	42	II	4.4
N39	health	35	II	4.2
N40	health	20	II	4.0
N41	health	29	II	4.2
N42	health	40	II	4.0
N43	health	23	II	4.0
N44	health	32	II	4.0
N45	health	26	II	4.0
N46	health	38	II	4.2
N47	health	31	II	4.0
BV1	BV	26	III	5.2
BV2	BV	28	III	5.4
BV3	BV	28	III	4.6
BV4	BV	29	III	5.4
BV5	BV	47	III	5.4
BV6	BV	49	III	5.4
BV7	BV	41	III	5.4

BV8	BV	21	Ⅲ	5.4
BV9	BV	79	Ⅲ	5.4
BV10	BV	32	Ⅲ	5.2
BV11	BV	27	Ⅲ	5.4
BV12	BV	38	Ⅲ	5.0
BV13	BV	29	Ⅲ	5.4
BV14	BV	26	Ⅲ	5.4
BV15	BV	38	Ⅲ	5.4
BV16	BV	42	Ⅲ	5.4
BV17	BV	47	Ⅲ	5.4
BV18	BV	41	Ⅲ	5.4
BV19	BV	37	Ⅲ	5.4
BV20	BV	50	Ⅲ	5.4
BV21	BV	72	Ⅳ	5.4
BV22	BV	47	Ⅲ	5.4
BV23	BV	50	Ⅲ	5.4
BV24	BV	23	Ⅲ	5.4
BV25	BV	28	Ⅲ	5.4
BV26	BV	52	Ⅲ	5.4
BV27	BV	26	Ⅲ	5.4
BV28	BV	46	Ⅲ	5.4
BV29	BV	45	Ⅲ	5.4
BV30	BV	42	Ⅲ	5.4
BV31	BV	29	Ⅲ	5.4
BV32	BV	34	Ⅲ	5.4
BV33	BV	20	Ⅲ	5.4

BV34	BV	53	Ⅲ	5.4
BV35	BV	72	Ⅲ	5.4
BV36	BV	47	Ⅲ	5.4
BV37	BV	34	Ⅲ	5.4
VVC1	VVC	47	Ⅲ	4.0
VVC2	VVC	24	Ⅳ	4.4
VVC3	VVC	37	Ⅲ	4.4
VVC4	VVC	35	Ⅲ	4.4
VVC5	VVC	44	Ⅲ	4.2
VVC6	VVC	41	Ⅲ	4.2
VVC7	VVC	35	Ⅲ	4.2
VVC8	VVC	22	Ⅲ	4.6
VVC9	VVC	47	Ⅲ	4.4
VVC10	VVC	27	Ⅲ	4.4
VVC11	VVC	26	Ⅲ	4.4
VVC12	VVC	27	Ⅲ	4.4
VVC13	VVC	48	Ⅲ	5.0
VVC14	VVC	25	Ⅲ	4.4
VVC15	VVC	38	Ⅲ	4.0
VVC16	VVC	31	Ⅲ	4.6
VVC17	VVC	22	Ⅲ	4.6
VVC18	VVC	28	Ⅲ	4.4
VVC19	VVC	46	Ⅲ	4.0
VVC20	VVC	26	Ⅲ	4.4
VVC21	VVC	30	Ⅲ	4.0
VVC22	VVC	29	Ⅲ	4.2

VVC23	VVC	48	III	4.4
VVC24	VVC	30	III	4.0
VVC25	VVC	40	III	5.0
VVC26	VVC	38	III	4.0
VVC27	VVC	43	III	4.2
VVC28	VVC	26	III	4.4
VVC29	VVC	34	III	4.6
VVC30	VVC	48	IV	4.6
VVC31	VVC	34	III	4.2
VVC32	VVC	25	III	4.6
VVC33	VVC	37	III	4.0
VVC34	VVC	28	IV	4.4
VVC35	VVC	20	III	4.2
VVC36	VVC	27	III	4.0
VVC37	VVC	21	III	4.0
VVC38	VVC	30	III	4.4
VVC39	VVC	45	IV	4.4
VVC40	VVC	30	III	4.4
VVC41	VVC	36	IV	4.2
VVC42	VVC	40	IV	4.6
VVC43	VVC	28	IV	4.4
VVC44	VVC	29	III	4.6
UU1	UU	27	III	5.0
UU2	UU	57	III	5.0
UU3	UU	29	III	5.0
UU4	UU	24	III	5.0

UU5	UU	38	Ⅲ	5.0
UU6	UU	46	Ⅲ	5.0
UU7	UU	51	Ⅲ	5.4
UU8	UU	25	Ⅲ	4.8
UU9	UU	42	Ⅲ	4.6
UU10	UU	35	Ⅲ	4.8
UU11	UU	27	Ⅲ	5.0
UU12	UU	38	Ⅲ	4.6
UU13	UU	27	Ⅲ	4.6
UU14	UU	31	Ⅲ	5.0
UU15	UU	27	Ⅲ	4.8
UU16	UU	52	Ⅲ	4.0
UU17	UU	29	Ⅲ	5.4
UU18	UU	49	Ⅲ	5.0
UU19	UU	47	Ⅲ	4.6
UU20	UU	25	Ⅲ	5.4
UU21	UU	34	Ⅲ	5.4
UU22	UU	25	Ⅲ	5.0
UU23	UU	32	Ⅲ	4.4
UU24	UU	36	Ⅲ	5.4
UU25	UU	26	Ⅳ	4.6
UU26	UU	38	Ⅲ	5.4
UU27	UU	29	Ⅲ	5.4
UU28	UU	40	Ⅲ	5.4
UU29	UU	44	Ⅳ	4.6
UU30	UU	31	Ⅲ	4.4

UU31	UU	38	Ⅳ	4.8
UU32	UU	49	Ⅲ	4.4
UU33	UU	52	Ⅲ	4.4
